# Fusarium head blight resistance in European winter wheat: insights from genome-wide transcriptome analysis

**DOI:** 10.1186/s12864-021-07800-1

**Published:** 2021-06-24

**Authors:** Maria Buerstmayr, Christian Wagner, Tetyana Nosenko, Jimmy Omony, Barbara Steiner, Thomas Nussbaumer, Klaus F. X. Mayer, Hermann Buerstmayr

**Affiliations:** 1 University of Natural Resources and Life Sciences, Austria, Department of Agrobiotechnology - IFA Tulln, Institute of Biotechnology in Plant Production, Konrad Lorenz Str 20, Tulln, Austria; 2 Helmholtz Zentrum München, Germany, PGSB Plant Genome and Systems Biology, German Research Center for Environmental Health, Neuherberg, Germany; 3 Helmholtz Zentrum München, Germany, Institut für Asthma- und Allergieprävention (IAP), Deutsches Forschungszentrum für Gesundheit und Umwelt (GmbH), Munich, Germany; 4 Helmholtz Zentrum München, Germany, Research Unit Environmental Simulation (EUS) at the Institute of Biochemical Plant Pathology (BIOP), Ingolstädter Landstraße 1, 85764 Neuherberg, Germany; 5 Helmholtz Zentrum München, Germany, Institute of Network Biology (INET), Ingolstädter Landstraße 1, 85764 Neuherberg, Germany; 6 Helmholtz Zentrum München, Germany, Institute of Environmental Medicine UNIKA-T, Technical University and Helmholtz Zentrum München, Augsburg, Germany

**Keywords:** *Triticum aestivum*, *Fusarium graminearum*, Sumai-3, *Fhb1*, *Qfhs.ifa-5A*, Cell wall modification, Terpene, NST1, RNA-seq

## Abstract

**Background:**

Fusarium head blight (FHB) is a devastating disease of wheat worldwide. Resistance to FHB is quantitatively controlled by the combined effects of many small to medium effect QTL. Flowering traits, especially the extent of extruded anthers, are strongly associated with FHB resistance.

**Results:**

To characterize the genetic basis of FHB resistance, we generated and analyzed phenotypic and gene expression data on the response to *Fusarium graminearum* (*Fg*) infection in 96 European winter wheat genotypes, including several lines containing introgressions from the highly resistant Asian cultivar Sumai3. The 96 lines represented a broad range in FHB resistance and were assigned to sub-groups based on their phenotypic FHB severity score. Comparative analyses were conducted to connect sub-group-specific expression profiles in response to *Fg* infection with FHB resistance level. Collectively, over 12,300 wheat genes were Fusarium responsive. The core set of genes induced in response to *Fg* was common across different resistance groups, indicating that the activation of basal defense response mechanisms was largely independent of the resistance level of the wheat line. *Fg*-induced genes tended to have higher expression levels in more susceptible genotypes. Compared to the more susceptible non-Sumai3 lines, the Sumai3-derivatives demonstrated higher constitutive expression of genes associated with cell wall and plant-type secondary cell wall biogenesis and higher constitutive and *Fg*-induced expression of genes involved in terpene metabolism. Gene expression analysis of the FHB QTL *Qfhs.ifa-5A* identified a constitutively expressed gene encoding a stress response NST1-like protein (TraesCS5A01G211300LC) as a candidate gene for FHB resistance. NST1 genes are key regulators of secondary cell wall biosynthesis in anther endothecium cells. Whether the stress response NST1-like gene affects anther extrusion, thereby affecting FHB resistance, needs further investigation.

**Conclusion:**

Induced and preexisting cell wall components and terpene metabolites contribute to resistance and limit fungal colonization early on. In contrast, excessive gene expression directs plant defense response towards programmed cell death which favors necrotrophic growth of the *Fg* pathogen and could thus lead to increased fungal colonization.

**Supplementary Information:**

The online version contains supplementary material available at 10.1186/s12864-021-07800-1.

## Background

Fusarium head blight (FHB), predominately caused by *Fusarium graminearum*, is one of the most destructive diseases of wheat and small grain cereals worldwide. Yield and quality losses can be devastating and mycotoxins produced by *Fusarium* pathogens compromise food and feed safety [1, 2].

FHB resistance is a quantitative trait, with more than 500 QTL reported in previous studies [3–5]. The Chinese spring wheat cultivar Sumai3 is among the most important and best characterized sources of FHB resistance and is the donor of the two major resistance QTL *Fhb1* and *Qfhs.ifa-5A* [6]. *Fhb1* was the first sequenced FHB resistance locus in wheat, yet the casual gene behind the *Fhb1* resistance remains unclear. A pore-forming toxin like (PFT) gene [7] and a histidine-rich calcium binding (HRC) protein [8, 9] have been proposed as candidate genes for *Fhb1.* The second resistance locus, *Qfhs.ifa-5A* was recently fine-mapped into the major effect QTL *Qhfs.ifa-5Ac* located on the centromere and the minor effect QTL *Qfhs.ifa-5AS* on the short arm of chromosome 5A [10].

*Fusarium* fungi colonize and invade wheat heads via open florets during anthesis, a complex and critical reproductive growth stage [11]. The fungi are biotrophic during infection, but once the host cell death is initiated, biotrophic growth is accompanied by necrotrophic intracellular colonization [12]. Production of the trichothecene toxin deoxynivalenol (DON) is specifically induced during colonization and may activate the transition from biotrophy to necrotrophy [13, 14].

Plants are constantly challenged by biotic and abiotic stresses. Hence, plants have evolved sophisticated surveillance and defense mechanisms that recognize and rapidly respond to potentially hazardous conditions [15]. Overall, transcriptomic studies have demonstrated that the response of wheat to *Fusarium* pathogens largely resembles stress defense reactions characteristic of most plant-pathogen interactions [16, 17]. These plant defense responses include induction of calcium ion influx, generation of reactive oxygen species (ROS), hypersensitive responses, phytohormone-related signaling, induction of pathogenesis-related genes, up-regulation of transcription factor activity, production of antioxidants and antimicrobial substances, detoxification, cell wall modification and cell wall fortification to name a few of the frequently reported defense responses [18–27]. Many of the induced genes showed expression changes in both resistant and susceptible genotypes suggesting, a broad range of basal defense responses [17, 28, 29]. However, genotype-specific gene expression and differences in transcript accumulation between genotypes have also been reported [17, 28, 30].

Plant defense depends on the fine-tuned and coordinated regulation of genes induced upon pathogen attack. It also depends on preexisting constitutive gene expression that provides a significant advantage to the host ahead of the infection. Constitutive defense includes physical and chemical barriers that efficiently impede fungal entry or slow down fungal progress once the fungus has penetrated the plant tissue. Because FHB infection starts inside the floral cavity, mechanisms reducing the likelihood of spores entering the spikelets (e.g. cleistogamous flowering, narrow opening width and short flower opening) increase FHB resistance [31, 32]. Anthers retained within the florets or trapped between the floral brackets are important fungal entry points and the preferred tissue at the onset of FHB infection [3]. Steiner et al. [10] found that *Qfhs.ifa-5A* has a strong effect on anther extrusion and FHB resistance suggesting a passive, constitutive resistance behind this QTL.

To date, studies on transcriptional response to *Fusarium* infection or DON infiltration have been restricted to a few wheat genotypes with contrasting resistance [16]. This is the first study that employs a large-scale analysis of gene expression and phenotypic data from 96 genotypes representing the European winter wheat gene pool and experimental lines with *Fhb1* and *Qfhs-ifa-5A* introgressions. The lines span a broad spectrum of FHB resistance from highly resistant to highly susceptible. We aimed to connect transcriptional patterns with FHB resistant and susceptible phenotypes. Previous studies on *Fhb1* or *Qfhs.ifa-5A-*associated resistance focused primarily on transcriptional profiling of near isogenic lines (NILs) [19, 22, 33–37]. Our panel included a small subset of lines carrying the resistance alleles *Fhb1* and *Qfhs.ifa-5A*. This allows for the comparison of expression profiles of resistance alleles in diverse genetic backgrounds and can assist in candidate gene identification.

### Experimental procedures

#### Plant material and field experiment for FHB resistance evaluation

The winter wheat panel consisted of 96 European genotypes, comprising elite cultivars, breeding lines and experimental lines. Fifteen of the experimental genotypes are offspring of ‘Sumai3’ or ‘CM-82036’ (Sumai3/Thornbird-S) that were phenotypically selected for their high resistance to FHB based on preceding experiments at IFA-Tulln, Austria*.* The panel was assessed for FHB severity in field tests at IFA Tulln in 2014 and 2015 as described by Michel et al. [38]. The wheat lines covered a broad range in FHB response from highly resistant to highly susceptible (Table S1).

#### Greenhouse experiment for RNA-sequencing

Plants were grown under controlled greenhouse conditions as described by Samad-Zamini et al. [35]. Per genotype, two replicates for *Fusarium*-treatment and one for control (mock-treatment) were planted with ten plants per pot using a randomized complete block design. Individual heads were inoculated at mid-anthesis. Per head, basal florets of six central spikelets were point-inoculated by pipetting 10 μl of either a *F. graminearum* (*Fg*) spore suspension (strain IFA65/66; 50,000 spores/mL) or distilled water (control heads) between palea and lemma to avoid wounding. Following treatments, heads were covered with plastic bags for 24 h to ensure high humidity for optimal fungal growth. Inoculations were done on consecutive days in February 2015 at approximately 10:00 AM to minimize confounding effects due to circadian gene expression.

Plant tissue of *Fg* and mock-treated spikelets (including rachis, excluding awns) were collected 48 h after inoculation (hai), immediately shock-frozen in liquid nitrogen and stored at − 80 °C. *Fg*-treated samples consisted of pools of five individual heads/replication (=pot). Mock-treated control samples consisted of pools of six individual heads/pot. RNA of pooled samples (100 mg) was extracted as described by Samad-Zamini et al. [35].

#### RNA sequencing, mapping and expression quantification with a dual RNA-seq approach

Two hundred eighty-eight Illumina HiSeq 2500 strand-specific RNA-seq libraries were sequenced in the 125 bp paired-end mode for the 96 wheat lines (three libraries per line) by GATC Biotech (Konstanz, Germany- now part of Eurofins Scientific). Adapters and low-quality reads were trimmed using Trimmomatic v.0.35 [39]. Data quality was assessed before and after trimming using FastQC [40]. The processed RNA-seq data was aligned using Hisat2 v.2.1.0 [41] to the reference containing the *Triticum aestivum* reference genome sequence IWGSCv1.0 [42] and the *Fhb1* region of the wheat cultivar CM-82036 (KU641029; GI: 1000816923 [36]), whose gene composition at *Fhb1* differs from the homologous locus in the Chinese Spring genome (*chr3Bfhb1*) [43]. The read pairs aligned to exonic regions were summarized per gene using featureCounts [44]. To recover read pairs that aligned to *Fhb1* and *chr3Bfhb1*, one or two to of the best alignments were kept for reads that mapped to multiple loci. Only reads uniquely mapped to a single locus were counted for the remaining non-*Fhb1* genes. The resulting raw count matrix was used as an input for the differential gene expression (DGE) analysis. The *chr3Bfhb1* locus was identified using sequence similarity search BLASTn of *Fhb1* against IWGSCv1.0 using an e-value threshold of 1.0e^− 30^. The number of read-pairs that aligned to *Fhb1* and *chr3Bfhb1* loci were assessed for each wheat line using SAMtools [45]. To test for batch effects, outliers and sample structure, preliminary data analysis was performed using variance stabilizing transformation with the R package DESeq2 [46].

#### Differential gene expression (DGE) analyses

DGE analysis was performed with the R-package DESeq2 [46]. The raw counts were filtered for minimum expression, in which genes with a minimum of 10 library-normalized counts present in at least five libraries were used for further analyses.

In order to compare expression responses to *Fusarium* infection in wheat lines with different FHB resistance levels, genotypes were grouped based on percentage of infected spikelets (PIS) 26 days after inoculation as follows: resistant (R, PIS < 20%), moderate resistant (MR, 20% < PIS > 65%) and susceptible (SUS, PIS > 65%). In addition, a highly resistant ‘Sumai3-derived’ (Sumai3, PIS < 6) group was formed comprising only Sumai3 and CM-82036 descendants carrying both, *Fhb1* and *Qfhs.ifa-5A* resistance loci. FHB resistance groups Sumai3, R, MR and SUS comprised 9, 18, 45, and 18 genotypes, respectively (Table S1).

DGE analyses were conducted as follows: i) DGE analyses between *Fg* and mock-treated samples were performed separately for each resistance group, for each genotype and across all genotypes to determine Fusarium responsive genes (FRGs), ii) Pairwise group comparisons were conducted for *Fg* and for mock-treated samples to determine genes differentially expressed (DEGs) between resistance groups, iii) DGE analyses for genotypes contrasting for the resistance allele at *Fhb1* and DGE analyses for genotypes contrasting for the resistance allele at *Qfhs.ifa-5A* were conducted to identify QTL-specific expressed genes.

The thresholds for differential expression was *p.*adjusted < 0.05, and |log_2_ expression Fold Change (log_2_FC)| > 1 for up and down-regulated genes. Functional analysis of annotated DEGs and the downstream gene set enrichment analysis (GSEA) were performed using R-packages GOstats and GSEABase [47].

## Results

### Gene expression analysis

**Fig. 1:**
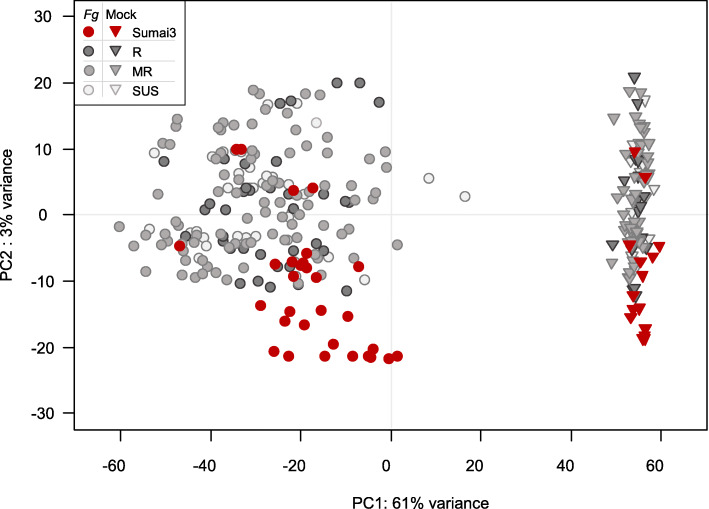
Principal component analysis of variance-stabilized-transformed read counts. Circles and triangles indicate *Fusarium graminearum* (*Fg*) and Mock treatment, respectively. Colors refer to the resistance groups Sumai3, Resistant (R), Moderate Resistant (MR), Susceptible (SUS)

Eighty-five percent of the total 7,311,347,144 RNAseq reads (429 Gbp) generated for this project passed the quality trimming and filtering as paired-end reads (3,112,438,347 read pairs; 4,917,846-24,111,765 read pairs per library with quality score Q30 94%). Of these reads, 2,936,689,266 (94.8%; 4,630,811-22,833,415) pairs per library were aligned to the reference sequences consisting of IWGSCv1.0 genome and *Fhb1* locus. In total, 106,582 genes (70,887 and 35,695 of high and low confidence, respectively) were expressed. Principal component analysis revealed that gene expression was mainly driven by the *Fg* versus mock-treatment, with the first principal component explaining 61% of the variation (Fig. 1).

### Fusarium induced changes in gene expression

**Fig. 2:**
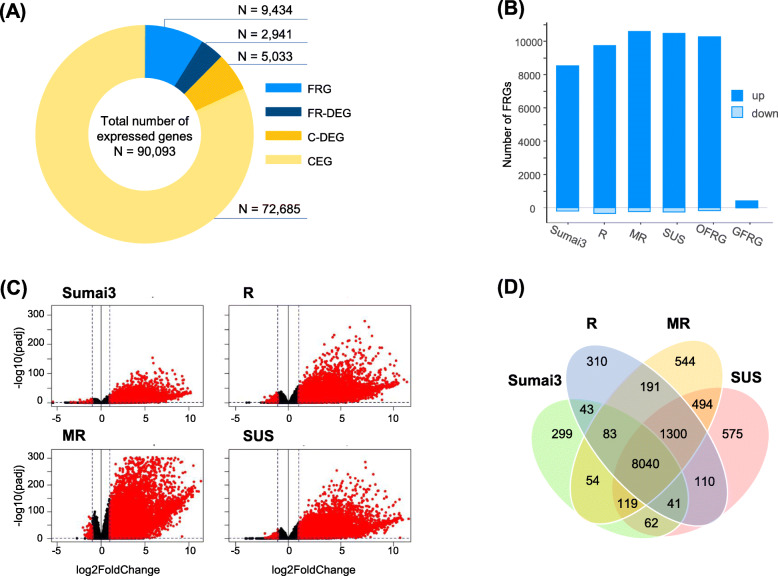
Summary of differential gene expression analyses. **A** Total number of expressed genes partitioned into Fusarium responsive genes (FRG) equally expressed across resistance groups, FRGs differentially expressed between resistance groups (FR-DEG), constitutively expressed genes (CEG) and CEGs differentially expressed between resistance groups (C-DEG). **B** Number of FRGs significantly up or downregulated 48 h after *Fusarium graminearum* inoculation for genotypes of resistance groups Sumai3 (Sumai3), Resistant (R), Moderate Resistant (MR) and Susceptible (SUS), and across all genotypes [Overall Fusarium responsive genes (OFRG)], and in each genotype [General Fusarium Responsive Genes (GFRG)]. **C** Volcano plots showing the distribution of the gene expression fold changes in each resistance group between *Fg* and mock treatment. Dots on the left and right sides of horizontal bold line represent downregulated and upregulated genes, respectively. Red dots represent significantly induced genes with |log_2_FC| > 1 (indicated by dashed horizontal line) and *p*-adjust ≤0.05 (indicated by dashed vertical lines). **D** Venn diagram showing shared and unique FRGs of resistance groups

Overall, 90,093 genes passed the minimum expression filtering step and were used for DGE analyses. Collectively, 12,375 genes (14%) were differentially expressed between *Fg* and mock-treatment in at least one analysis (Fig. 2A). Within the Sumai3, R, MR and SUS resistance groups 8741, 10,118, 10,825 and 10,741 wheat genes were Fusarium responsive (FR), respectively (Fig. 2B, Table S2), with most genes being up-regulated (~ 95%) (Fig. 2C). Overall, 8040 (65.5%) genes were induced in all resistance groups. Additionally, 1300 (10.6%) FRGs were shared by the R, MR and SUS group, but not by the Sumai3 resistance group (Fig. 2D).

**Fig. 3:**
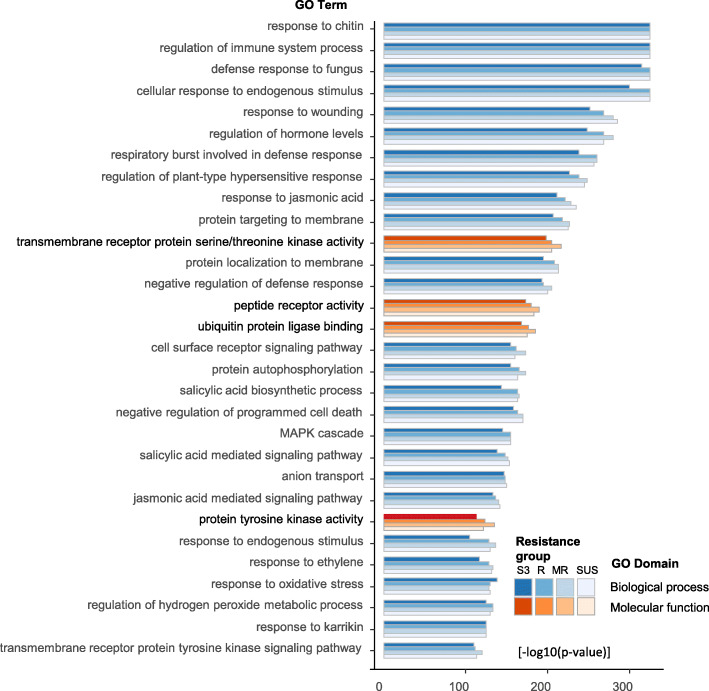
Top 30 gene ontology (GO) terms enriched for genes significantly upregulated 48 h after *Fusarium graminearum* inoculation for individual resistance groups Sumai3 (S3), resistant (R), moderate resistant (MR) and susceptible (SUS). GO terms are ranked according the log10(*p*-value) and filtered by odds ratio ≥ 3 between expected and matched gene counts. For additional information see Table S3

Gene ontology (GO) analysis revealed enrichment of the FRGs of individual resistance groups for over 600 biological processes (BP) and over 150 molecular functions (MF) (Table S3). BP terms were largely involved in metabolic process, biological regulation, response to stimulus, cellular process and immune system process. Response to chitin, defense response to fungus, response to endogenous stimulus, regulation of immune system process, respiratory burst involved in defense response, regulation of plant-type hypersensitive response, response to and regulation of hormone levels and signaling were the top enriched GO terms (Fig. 3, Table S3). MFs were enriched for terms associated with catalytic activity, molecular transducer activity and binding. Transmembrane receptor protein serine/threonine kinase activity, peptide receptor activity, protein tyrosine kinase activity, glutathione transferase activity, ubiquitin protein ligase binding, carbohydrate and calmodulin binding were key MF components (Fig. 3, Table S3).

In total, 422 (414 up-regulated, 8 down-regulated) of the FRGs were induced in all genotypes and were considered as general FRGs (GFRGs) (Fig. 2B, Tables S2). Over 25% of the GFRGs were functionally characterized as protein-like kinase, receptor-like proteins, and receptor-like protein-kinase, indicating general activation of signaling pathways that initiate plant immune and defense responses. Among the most highly upregulated GFRGs were DUF538 family proteins, cytochrome P450, WRKY transcription factors, glycosyltransferases, receptor-(like)-kinases and pathogenesis-related proteins (Table S2, Table S4).

### Differences in gene expression between resistance groups

**Fig. 4:**
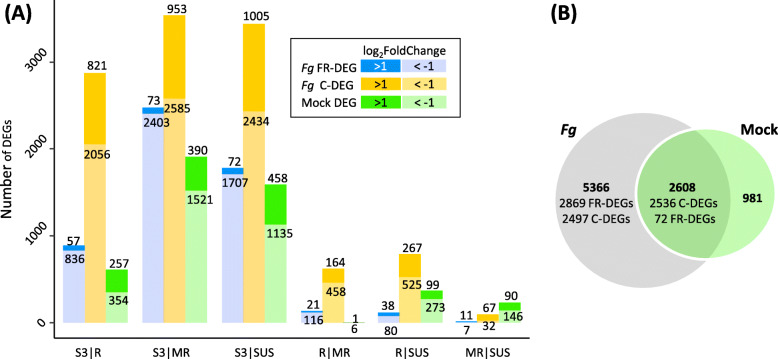
**A** Number of genes significantly up- or down regulated (log_2_FoldChange | > 1|, p-adjust ≤0.05) in the pairwise group-comparison between resistance groups Sumai3 (S3), resistant (R), moderate resistant (MR), susceptible (SUS) 48 h after *Fusarium graminearum* (*Fg*) and after Mock treatment (Mock_DEG). *Fg* treated samples: *Fg*_FR-DEG: gene significantly differentially expressed both in response to *Fg* relative to Mock and between compared groups; *Fg*_C-DEG: gene constitutively differentially expressed between compared groups. For detailed information see Table S5. **B** Venn diagram showing shared and unique DEGs after *Fg* and Mock treatment

Collectively, 7974 and 3589 genes were differentially expressed between the resistance groups after *Fg* and mock-treatment, respectively (Table S5). Between groups, most DEGs under mock-treatment (75%) were also differentially expressed under *Fg* infection (Fig. 4B).

### Fusarium responsive DEGs

Generally, the number of induced genes and the respective transcriptional abundance increased with susceptibility of the genotypes under investigation. The Sumai3 resistance group had 16 to 24% fewer FRGs than the resistance groups R, MR and the SUS (Fig. 2B). FR gene expression was significantly different between the Sumai3 group and the R, MR and SUS groups for 893, 2476 and 1707 FRGs, respectively. Expression profiles were most similar between the resistance groups R|MR, R|SUS and MR|SUS, amounting to 137, 118 and 18 FR-DEGs between groups (Fig. 4A, Table S5).

### Constitutive DEGs

Approximately 86.3% (77,718) of all expressed genes were constitutively expressed genes (CEG) and showed no differences in expression level between mock and *Fg*-treated samples. Overall, 5033 of the CEGs were significantly differentially expressed between resistance groups (C-DEGs), thereby potentially conferring passive (constitutive) disease resistance (Fig. 2A). Again, the Sumai3 derivatives were markedly different from all the other groups.

Over 90% of the FR-DEGs and 70% of the C-DEGs had significantly higher expression levels in the R, MR or SUS groups relative to the highly resistant Sumai3 derivatives (Fig. 4). Two-thirds of the DEGs between R|MR and R|SUS had higher expression levels in the more susceptible groups MR and SUS, respectively (Table S5). We grouped genes according to their functional description and compiled summary statistics of the identified FRGs, FR-DEGs and C-DEGs (Table S4). FR-DEGs were dominated by calcium-binding protein, germin-like protein, specific transcription factors (WRKY, ethylene responsive transcription factor, NAC, Myb), and genes involved in detoxification (UDP-glycosyltransferase, glutathione S-transferase, protein detoxification, drug resistance ATP-binding cassette (ABC) transporter) and cell wall fortification (phenylalanine ammonia-lyase, agmatine coumaroyl transferase, blue copper protein, laccase). C-DEGs were overrepresented by NBS-LRR genes, F-box related proteins and transposable elements including retrotransposons and retrovirus related transposons (Table S4, col. F&G).

**Fig. 5:**
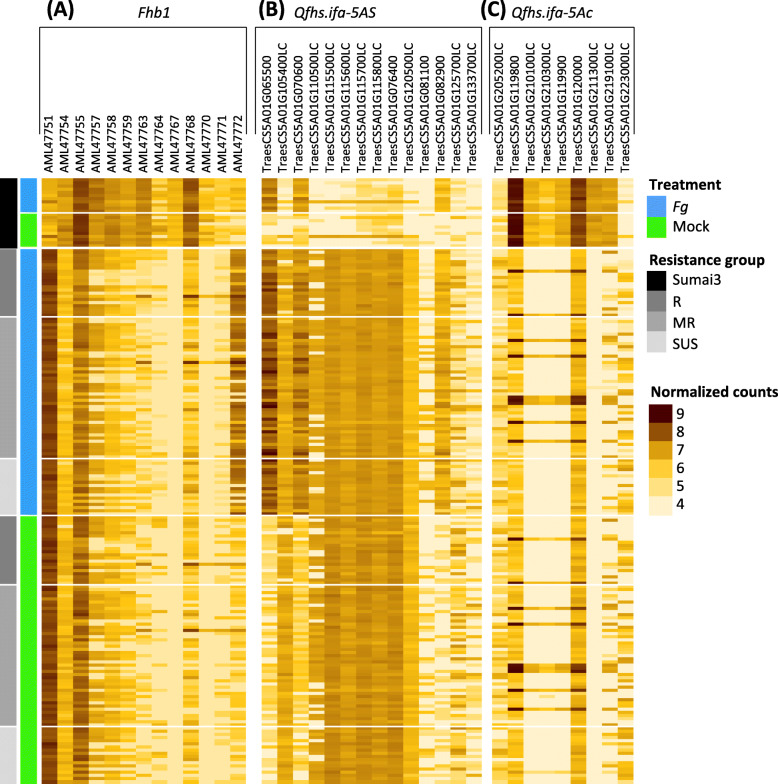
Expression profiles of genes in the individual wheat lines (rows) within the QTL regions **A** *Fhb1* contig, **B** *Qfhs.ifa-5AS*, and **C** *Qfhs.ifa-5Ac*. Only genes are shown that are significantly differentially expressed between Sumai3 (carrier of the resistance allele) and non-Sumai3 (no resistance allele) groups

### GSEA of genes differentially expressed between resistant groups

To explore the functions of the genes differentially expressed between the resistance groups, we performed GSEA of DEGs obtained by pairwise comparison of groups and joint comparison of ‘Sumai3-derived’ to ‘non-Sumai3-derived’ genotypes.

### GSEA of FR-DEGs

#### Sumai3 derivatives versus European gene-pool

FR-DEGs of the Sumai3 group were overrepresented by up-regulated genes annotated as BP GO terms involved in terpene and phosphate-metabolism and protein phosphorylation (Table S6.2). In contrast, GO term analysis of FRGs highly expressed and enriched in the R, MR and SUS relative to Sumai3 group revealed diverse BPs, with over 300 sub-categories largely associated with response to stimulus, biological regulation, and cellular-, immune system-, metabolic- and development processes. Response to nitrogen compound, respiratory burst involved in defense response, response to chitin, immune system process, cell communication and regulation of hormone levels were among the most enriched terms (Table S6.2). FR-DEGs involved in UDP-glucosyl and UDP-glucose transferase activity and in peptide and transmembrane signaling receptor activity were among the most enriched MF terms. These genes were highly upregulated in the R, MR and SUS resistance groups compared to the Sumai3 group.

#### Group comparisons within European gene-pool

Genes more highly up-regulated by the MR and SUS groups than by the R group were enriched for catalytic activities and metabolic processes (Table S6.2). The R group demonstrated enrichment for genes involved in anatomical structure development and developmental processes involved in reproduction, whereas the SUS group was overrepresented by GO terms involved in metabolic processes.

### GSEA of constitutively expressed C-DEGs

#### Sumai3 derivatives versus European gene-pool

The Sumai3 group was enriched for genes associated with protoxylem development, plant-type secondary cell wall, triterpenoid biosynthesis and glycerophosphate shuttle for C-DEGs after *Fg*-treatment (Table S6.3, Figure S1). Terpene, terpenoid and hemicellulose metabolic processes and terms associated with cell wall biogenesis were overrepresented after mock-treatment in Sumai3 compared to the non-Sumai3 or SUS groups (Table S6.4). The non-Sumai3 groups were enriched for functional processes contributing to immune and defense response.

#### Group comparisons within the European gene-pool

Compared to the SUS group, differently expressed genes in the R group were enriched and up-regulated for GO terms associated with reproduction and anatomical structure development (anther dehiscence, pollen sperm cell differentiation, cell wall modification involved in abscission) and pectin catabolic processes. C-DEGs upregulated in the SUS and MR groups were more abundant and diverse and were enriched for 26 and 31 functional categories of GO BPs and MFs, respectively (Table S6.3). The most highly enriched BP terms were associated with lipid transport, chromatin organization (regulation of chromatin assembly, regulation of methylation-dependent chromatin silencing, histone acetylation), down-regulation of endopeptidase and hydrolase activity, downregulation of proteolysis and protein metabolic process. The most highly enriched MFs were involved with lipid binding, enzyme regulator activity, and pectin esterase-, peptidase- and cysteine-type endopeptidase inhibitor activity.

### Expression analyses of genes located in the *Fhb1*, *Qfhs.ifa-5AS* and *Qfhs.ifa-5Ac* QTL regions

Marker analyses confirmed the presence of the resistance alleles for either *Fhb1* or *Qfhs.ifa-5AS* and *Qfhs.ifa-5Ac* or for all three QTLs in two, two and nine of the 15 Sumai3 descendent genotypes, respectively (Table S1). Genes located within the QTL intervals were analyzed for differential transcription abundance between treatments and genotypes by contrasting for the respective resistance QTL.

### Differentially expressed genes in the *Fhb1* QTL interval

The *Fhb1* QTL interval comprises 28 candidate genes [43], of which 13 revealed significant differential expression between lines contrasting for *Fhb1* (Table 1, Fig. 5). One of the genes, a GDSL lipase acylhydrolase (AML47772), was responsive to *Fg* and downregulated in non-*Fhb1* carriers. The other 12 candidate genes showed constitutive expression changes with predominantly higher transcript levels in Sumai3-derivatives harboring *Fhb1*. PFT (AML47770) and HRC (AML47768), previously reported to solely confer resistance to fungal spreading [7–9] showed higher, treatment-independent expression patterns in ‘*Fhb1* genotypes’. The highest and most distinct transcript abundance difference between *Fhb1* and non-*Fhb1* carriers was observed for a Terpene synthase (AML47767) with exclusive expression in ‘*Fhb1* genotypes’.

**Table 1:** Differentially expressed genes between genotypes contrasting for the resistance allele *Fhb1* or *Qfhs.ifa-5A*

Gene ID^a^	bp position start	Human-Readable-Description	Allele^b^	Treatm^c^
			Log_2_FC	Log_2_FC
***Fhb1***				
AML47751	8,140,000	Uncharacterized Protein	-3.4	
AML47754	8,200,000	Glycosyltransferase HGA-like	2	
AML47755	8,220,000	Leucyl-tRNA synthase	1.3	
AML47757	8,260,000	Alanyl-tRNA synthase	2	
AML47758	8,280,000	Uncharacterized Protein	2.7	
AML47759	8,300,000	PAP fibrilling domain containing protein	3	
AML47763	8,360,000	Oxidoreductase NAD-binding domain	4.1	
AML47764	8,380,000	Terpene synthase	3.3	
AML47767	8,440,000	Terpene synthase	5.6	
AML47768	8,460,000	Histidine-rich calcium-binding-protein gene	3.3	
AML47770	8,500,000	Agglutinin /Pore-forming toxin-like gene (PFT)	4.4	
AML47771	8,520,000	E3 ubiquitin-protein ligase	1.4	
AML47772	8,540,000	GDSL Lipase acylhydrolase	-2.9	4.6
***Qfhs.ifa-5AS***				
TraesCS5A01G065500	71,397,157	Glycosyltransferase	-1.3	6.4
TraesCS5A01G105400LC	77,338,937	Retrovirus-related Pol polyprotein from transposon TNT 1-94	-3.9	
TraesCS5A01G070600	79,152,530	Zinc finger protein, putative	-1	4.4
TraesCS5A01G110500LC	85,151,081	Penicillin-insensitive murein endopeptidase	-3.5	
TraesCS5A01G115500LC	92,180,537	Transposon Ty3-G Gag-Pol polyprotein	-4	
TraesCS5A01G115600LC	92,181,290	Transposon Ty3-G Gag-Pol polyprotein	-3.7	
TraesCS5A01G115700LC	92,182,337	Pol polyprotein	-3.7	
TraesCS5A01G115800LC	92,183,426	Ty3-gypsy retrotransposon protein	-3.1	
TraesCS5A01G076400	92,184,572	Retrotransposon protein, putative, unclassified	-3.2	
TraesCS5A01G120500LC	103,107,652	NADPH--cytochrome P450 reductase	-3.4	
TraesCS5A01G081100	104,147,359	cation/H+ exchanger 18	-3.2	
TraesCS5A01G082900	108,577,776	Receptor-like protein kinase	-1.1	4.8
TraesCS5A01G125700LC	109,629,216	Large proline-rich protein BAG6	-2.8	
TraesCS5A01G133700LC	119,060,876	Zinc finger (CCCH-type) family protein / RNA recognition motif (RRM)-containing protein	-2.6	
***Qfhs.ifa-5Ac***				
TraesCS5A01G205200LC	246,821,246	Retrovirus-related Pol polyprotein from transposon TNT 1-94	-1.5	
TraesCS5A01G119800	253,592,929	3′(2′),5′-bisphosphate nucleotidase 1	3	
TraesCS5A01G210100LC	253,593,703	Transposon Ty3-G Gag-Pol polyprotein	3.2	
TraesCS5A01G210300LC	253,595,868	Gag-pol polyprotein	3	
TraesCS5A01G119900	253,596,702	Transposon Ty3-I Gag-Pol polyprotein	3.2	
TraesCS5A01G120000	253,604,999	Pyruvate dehydrogenase E1 component alpha subunit	2.4	
TraesCS5A01G211300LC	257,282,460	Stress response NST1-like protein	7.3	
TraesCS5A01G219100LC	268,595,903	Protein FAR1-RELATED SEQUENCE 3	2.5	
TraesCS5A01G223000LC	274,993,878	Bifunctional glutamine synthetase adenylyltransferase/adenylyl-removing enzyme	-3.6	

^a^Only genes located within the QTL intervals of *Fhb1*, *Qfhs.ifa-5AS* and *Qfhs.ifa-5Ac* and p-adjust ≤ 0.05 |log_2_FC| > 1 are listed

^b^Positive log_2_FC indicate higher gene expression in lines carrying the resistance allele

^c^Treatment, positive log_2_FC indicate higher gene expression in *Fg* inoculation samples compared to mock-treatment

### Differentially expressed genes in the *Qfhs.ifa-5A* QTL interval

Within the *Qfhs.ifa-5AS* (70.7–119.9 Mbp) and *Qfhs.ifa-5Ac* (245.9–290.0 Mbp) regions, 216 and 108 genes were expressed, respectively. Fourteen genes within the *Qfhs.ifa-5AS* and nine genes within the *Qfhs.ifa-5Ac* interval were differentially expressed between groups contrasting for the resistance allele (Table 1, Fig. 5). Three genes within the *Qfhs.ifa-5AS* region, characterized as a glycosyltransferase (TraesCS5A01G065500), a zinc finger protein (TraesCS5A01G070600) and a receptor-like protein kinase (TraesCS5A01G082900), were *Fg*-induced, and were more highly up-regulated in the non-Sumai3 group. All remaining DEGs were constitutively differentially expressed. More than half of the DEGs comprised transposon-, retrotransposon-, or retrovirus-related proteins. DEGs within the *Qfhs.ifa-5AS* interval had higher expression levels in the group lacking the resistance allele. In contrast, higher transcript abundance was associated with the presence of the resistance allele for the centromeric QTL *Qfhs.ifa-5Ac*. Only the two genes flanking the *Qfhs.ifa-5Ac* region had higher expression levels in the non-Sumai3 derived lines. The highest expression ratio (log_2_FC = 7.3) was observed for the stress response NST1-like protein (TraesCS5A01G211300LC) located within the *Qfhs.ifa-5Ac* interval at 257,282,460 bp, next to the centromere. TraesCS5A01G211300LC was constitutively expressed in all lines containing the Sumai3 allele and not expressed in lines lacking the resistant allele.

## Discussion

We analyzed 96 genotypes, including 15 lines with Sumai3 in their pedigree and 81 European cultivars and breeding lines with a broad variation in FHB resistance. We sampled probes for RNAseq analyses 48 hai – a time point at which the majority of the transcripts are induced by the pathogen and is thus highly informative for expression analysis [22, 48]. At around 48 hai the biotrophic lifestyle of the *Fg* pathogen at the advancing hyphal front is already complemented by a necrotrophic lifestyle feeding on dead tissues [12, 27]. This joint action of both lifestyles requires a tailored and coordinated host defense strategy, as some host defense responses against biotrophs, e.g., programmed cell death (PCD), confer susceptibility to necrotrophs [49].

### *Fg*-induced transcriptional reprogramming

*Fg* inoculation initiated an extensive transcriptional reprogramming suggesting a highly complex host-pathogen interaction. Over 12,300 FRGs were identified, most of which were up-regulated (Fig. 2A). Around two-thirds of the FRGs were induced in all resistance groups showing that resistant and susceptible genotypes activated similar defense response mechanisms (Fig. 2B). However, approximately 25% of the FRGs differing in expression between resistance groups demonstrated an association between higher expression and increased susceptibility. This result corroborates with Pan et al. [28], Biselli et al. [26], and Wang et al. [17], in which the majority of the *Fg*-induced genes were shared by all wheat genotypes, with higher expression levels typically found in more susceptible lines. Consistent with earlier transcriptional studies, key components of Fusarium response fell into categories and pathways associated with defense responses, such as increased calcium influx, bursts of intracellular ROS, activation of transcription factors, regulation of immune system process, regulation of plant-type hypersensitive response, response to and regulation of hormone levels, accumulation of pathogenesis-related proteins, proteins involved in detoxification, cell wall reinforcement and lignin biosynthesis [16, 17, 21, 27, 28].

### Differences in gene expression between resistance groups

Aiming to identify expression patterns that discriminate genotypes according to their resistance level, we conducted pairwise group comparisons. DGE analysis between Sumai3 and the non-Sumai3 groups R, MR, SUS yielded ten and five times more induced FR-DEGs and constitutive C-DEGs, respectively, than group comparisons between R|MR and R|SUS (Fig. 4A). Members of the resistance group Sumai3 are closely related European genotypes with Sumai3 introgressions, while the non-Sumai3 genotypes represent diverse European cultivars and breeding lines which were grouped based on resistance to FHB only (Table S1). The non-Sumai3 groups are not only more genetically different from the Sumai3 group, but they also have a broader genetic and phenotypic within-group variance. Response mechanisms among group members may thus be more diverse. As such, response signals found in one or few lines may remain undetected by statistical analysis, leading to the lower number of DEGs found between the R, MR and SUS resistance groups in comparison to those between the Sumai3 group and the R, MR or SUS groups (Fig. 4A).

### Pathogen recognition – the first player in the pathogen-host interaction

Receptor-like kinases (RLKs) and nucleotide-binding leucine-rich repeat (NLRs) gene families play crucial roles in pathogen recognition and represent the first layer in downstream activation of plant defense mechanisms [50–52]. RLKs and NLRs were the largest group of *Fg* induced genes and the majority of these genes were equally induced across resistance groups (Tables S2, S4). About a quarter of the RLKs/NLRs were exclusively induced in the non-Sumai3 groups. RLKs/NLRs that were differentially expressed between groups showed higher expression levels in the more susceptible groups. This suggests, that in the more resistant lines, particularly in the Sumai3 lines, pathogen recognition may have already occurred before 48 hai, or constitutive resistance mechanisms against pathogen infection led to reduced induction of RLKs/NLRs. The pattern of moderate response associated with resistance and an excessive response associated with susceptibility was observed in most of the downstream activated defense reactions and may be a direct consequence of enhanced RLK/NLR activity in the more susceptible genotypes.

### Downstream activation of plant defense mechanism

*Ca*^*2+*^ *and reactive oxygen species (ROS) as signatures of initial direct plant defense response*

*Calcium signaling:* Calcium (Ca^2+^) is a universal second messenger involved in virtually all biotic and abiotic stress responses. Upon perception of stress signals by the membrane receptors Ca^2+^ influx will be induced within seconds. This transient elevated cytosolic Ca^2+^ concentration activates calmodulin and Ca^2+^ dependent protein kinases, nitric oxide and ROS that affect the function of many genes including hormone signaling and transcription factors, which control stress regulated genes [53–55]. We identified over 200 genes with Ca^2+^-binding domains and calmodulin-like protein families upregulated after *Fg* treatment (Table S2 col. AH, Table S4 col. AA). These genes may be critical for adequate defense response, since their upregulation is among the earliest events during the *Fg*-host interaction [27].

*ROS signaling:* ROS, which are partially reduced or activated derivatives of molecular oxygen, are rapidly produced and accumulated in the early phase of the pathogen-host interaction leading to the ROS-mediated oxidative burst [56]. ROS signaling is, amongst others, implicated in pathogen defense, plant hormone response, and systemic acquired resistance when kept in balance, while excess ROS is toxic to plant cells causing cell death [57–59]. Although PCD is a good strategy to ward off biotrophic pathogens, it increases susceptibility once *Fg* has switched to the necrotrophic lifestyle. ROS accumulation needs to be counterbalanced by antioxidants to maintain redox homeostasis [60]. Khaledi et al. [61] suggest that a rapid induction of ROS in combination with a rapid induction of antioxidant enzyme activity increases resistance against FHB. We found an activation of 217 predominately upregulated enzymatic antioxidant genes among which 185 were glutathione-S-transferases (GSTs) (Table S2 col. AI, Table S4 col. AE). Per resistance group, 31–40 GSTs were among the top 10% of genes with the highest fold change in expression after *Fg* treatment. An induction of numerous GSTs following *Fg* treatment was observed by Pan et al. [28] and in accordance to our results GSTs were up-regulated in FHB resistant and susceptible genotypes. GSTs are antioxidants, which help to limit PCD [62, 63], and participate in DON detoxification by the formation of DON-glutathione conjugates [20, 64]. GSEA analysis revealed ‘Respiratory burst involved in defense response’ as one of the most highly enriched GO terms in all resistance groups (Fig. 4) and underscores the general importance of oxidative burst in *Fg* defense response. Genes contributing to ROS and PCD were more highly upregulated and enriched in non-Sumai3 genotypes relative to Sumai3 lines (Tables S6.1). Since lower levels of early defense responses (RLK/NLR, Ca^2+^, ROS) are associated with increased FHB resistance we assume that the fate of the *Fg*-host interaction will be shaped at or before the onset of the infection and likely depends on constitutive defense mechanisms. The idea that constitutive gene expression may be critical for triggering adequate defense responses is furthermore supported by the few isolated FHB resistance genes. All three cloned FHB resistance genes are constitutively expressed and related to early defense response, with *Fhb1* encoding a putative histidine-rich calcium-binding protein [8], *Fhb7* encoding a glutathione S-transferase [65] and *QFhb.mgb-2A* predicted to encode a wall-associated receptor-like kinase [66].

### Host defense responses to limit Fusarium spread

#### Mycotoxin detoxification and cell wall modifications as important components for impeding fungal spread

*Host responses to mycotoxins accumulation:* Members of the *Fg* species complex produce trichothecene type B toxins that are secreted from the fungal hyphae tip [67]. These mycotoxins are virulence factors that determine the aggressiveness of the Fusarium pathogen and are essential for fungal penetration of the rachis and further spread within the wheat spike [13]. DON triggers ROS production and – depending on the level of ROS accumulation – initiates PCD promoting necrotrophic fungal growth and disease development [14]. Plants can reduce DON toxicity through chemical modification into less toxic DON-3-glucoside by uridine-diphosphate glycosyltransferases (UGTs) or the formation of DON-glutathione conjugates by GSTs and through toxin efflux by ABC transporters [36, 64, 68–71]. In our study, 51 UGTs, 179 GSTs and 119 ABC transporters were upregulated after *Fg* treatment (Table S2 col. AM). The majority of these genes (80%) were upregulated in all resistance groups with equal or lower levels of gene expression in Sumai3 compared to the non-Sumai3 groups. Differences in gene expression were minimal between the groups R, MR and SUS despite distinct resistance levels, showing that detoxification is an important defense response but has limited power to fully compensate for the higher DON accumulation in the more susceptible groups.

*Induced cell wall modifications and constitutive differences in cell wall components affect defense response:* Gunnaiah and Kushalappa [72] and Gunnaiah et al. [73] found cell wall thickening together with the antimicrobial and antioxidant properties of induced phenylpropanoid and flavonoid metabolites as the main resistance mechanisms of the Sumai3 cultivar. *Fg* inoculation increased lignin and hemicellulose signals in Sumai3, while signals related to oxidative stress were present in the susceptible cultivar only [74]. Kang and Buchenauer [75] likewise observed that lignin accumulated faster and more intensely in inoculated wheat spikes of resistant cultivars. They concluded, that the combination of cell wall thickness, cell wall composition and lignification determine host resistance to fungal spread within the spike. Our data suggest a general importance of cell wall thickness and lignification, since GO terms associated with cell wall thickening or defense response by callose deposition and GO terms linked to phenylpropanoid metabolic process, particularly lignin metabolic, lignin biosynthetic, and lignin catabolic processes were strongly enriched in all resistance groups (Table S3). Genes described as laccase, blue copper protein or agmatine coumaroyltransferase-2 were strongly induced and belonged to the top 10% FRGs with the highest log_2_FC in all resistance groups (Table S4). Laccase and blue copper protein together with dirigent protein and peroxidase are known mediators of lignin polymerization [76, 77] and contribute to increased defense-induced lignification and lignin accumulation in secondary cell walls [37, 78]. Transient silencing of laccase *TaLAC4* resulted in increased susceptibility leading to *Fg* spread within the wheat spike, while non-silenced NILs had thickened cell walls and higher total lignin content [37]. Lignin is one of the most persistent and difficult plant compounds to be decomposed by fungi [79, 80]. Hence, cell wall reinforcement via lignification provides both a physical barrier against pathogen invasion and chemical protection against fungal cell wall degrading enzymes. When comparing levels of constitutive gene expression between groups, we found enrichment in the Sumai3 relative to the non-Sumai3 or SUS groups for genes related to cell wall biogenesis, plant-type secondary cell wall and associated terms (Table S6). We assume, that the secondary cell wall composition of Sumai3 lines differs from non-Sumai3 lines. This difference possibly provides preconditional defense response that may be critical for initiating a coordinated though less intense activation of defense processes in the Sumai3 compared to the non-Sumai3 groups.

Since less than 20% of the induced genes were significantly more highly expressed in Sumai3 compared to non-Sumai3 genotypes, comparably fewer GO terms were enriched for DEGs that were more highly upregulated in the Sumai3 group (Figure S2). The majority of the GO terms that were more strongly induced in the Sumai3 group belonged to terpene or terpenoid metabolic processes and terpene synthase activity and were also found in the mock treated samples (Table S6, Figure S1). For example, a gene encoding terpene synthases (TraesCS5B01G01480) was constitutively expressed and showed the second highest positive fold change (log_2_FC = 14.7) among all DEGs between the Sumai3 and non-Sumai3 groups (Table S5.2). Terpenoids constitute the most chemically and structurally diverse class of plant secondary metabolites [81], many of which have antimicrobial and antioxidant properties and are involved in plant defense signaling, ROS scavenging and reinforcement of physical barriers [82, 83]. Among metabolomic studies terpenoids were found to be the third most frequently encountered secondary metabolites that were implicated in *Fg* defense in wheat and barley [20, 83–85]. Terpenoids were positively associated with FHB resistance in the cultivar Sumai3 [72, 73]; a terpene-synthase located within the *Fhb1* contig was constitutively expressed only in NILs that carried the *Fhb1* resistance allele [43].

### *Fhb1-* and *Qfhs.ifa-5A-*specific gene expression

#### The *Fhb1* enigma – expression patterns of 96 wheat genotypes identify several *Fhb1*-associated candidates

To date, four conflicting studies have reported the isolation of the gene controlling resistance to fungal spread at the *Fhb1* locus. Rawat et al. [7] pinpointed a PFT gene as the major contributor of the *Fhb1-*mediated resistance*.* Su et al. [9] and Li et al. [8] suggested a deletion in the HRC gene as the responsible mutation behind the *Fhb1*-mediated resistance. However, the two studies disagreed on the mode of action being either the result of a recessive loss-of-function mutation [9] or a functionally novel allele actively conferring resistance [8]. Moreover, recently, Paudel et al. [86] claimed that HRC acts as suppressor of *WFhb1–1*, which they suggested as the functional component of *Fhb1*. *WFhb1–1* is located outside the QTL interval, but the deletion in HRC inactivates its suppression and results in the ‘resistant HRC allele’ [86].

To further elucidate this puzzling locus, we studied gene expression of all 28 genes located in the *Fhb1* contig, including PFT (AML47770) and HRC (AML47768). Thirteen of the candidates, were constitutively differentially expressed in presence or absence of *Fhb1*, but only a GDSL Lipase acylhydrolase (AML47772) showed constitutive- and pathogen-dependent expression patterns (Fig. 5, Table 1). Our results largely agree with a previous dense time-course study of *Fhb1* candidate gene expression in two NILs [36]. We found exclusive expression in *Fhb1* carrier only for a Terpene synthase (AML47767) suggesting a special role of this gene in *Fhb1-*mediated resistance. In contrast, HRC was expressed in many genotypes with varying resistance levels, albeit to a much weaker extent in lines without the *Fhb1* resistance allele. This expression pattern is inconsistent with the proposed susceptibility factor at HRC [9, 86].

#### Differential gene expression analysis reveals a stress responsive NAC secondary wall thickening-promoting factor1 (NST1) like protein as a potential candidate for *Qfhs.ifa-5Ac-*mediated resistance

Centromeric and interstitial regions are known to be rich in transposable elements (TEs) [87]. This might explain the high proportion of TE-like proteins (transposon-, retrotransposon-, or retrovirus-related proteins) among DEGs identified across both QTL (Table 1). Although long considered ‘junk’ DNA, it is now acknowledged that TEs are important sources of binding sites for transcription factors; they can mobilize and respond to stress elicitors, alter expression of nearby genes and affect gene methylation and epigenetic adaptation [88]. TE-like protein homologs across *Qfhs.ifa-5A* loci were all constitutively differentially expressed. Two Gypsy-like retrotransposons were upregulated in response to DON in roots of the Sumai3 descendent CM82036 (a carrier of the resistance alleles at *Qfhs.ifa-5A* and *Fhb1*) supporting an active defense response [89].

Among all DEGs across both 5A QTL, only a stress responsive NST1-like protein (TraesCS5A01G211300LC) clearly discriminated between the resistant and susceptible haplotypes, being exclusively and constitutively highly expressed in the presence of the resistance allele at TraesCS5A01G211300LC (Table 1, Fig. 5). Genetic experiments on the model plants *Arabidopsis thaliana* and *Medicago truncatula* revealed the NAC transcription factor NST1 as a key regulator for the biosynthesis of plant-type specific secondary cell wall thickening genes in anther endothecium cells [90–94]. Anthers are considered as susceptibility factors when retained inside the floret. *Qfhs.ifa-5Ac* was found to simultaneously increase anther extrusion and FHB resistance [10], which is in line with the constitutive expression of NST1 in *Qfhs.ifa-5Ac* carriers. NST1 is required for anther dehiscence [94], however, it is unclear if NST1 affects the process of anther extrusions as well, which involves lodicule swelling for successful flower opening and filament elongation. An ectopic expression of NST1 was observed in various tissues, including filaments of stamens and the base of carpels leading to striated tracheary element-like structures in epidermal cells [94]. After dehiscence and anther extrusion, filaments remain fully rigid for a short time. Whether NST1 induced ‘tracheary’ structures affect rigidity of filaments that may help push the anthers out of the floret needs further investigation.

*Qfhs.ifa-5A* primarily confers resistance to fungal entry and early disease development (type 1 resistance), assessed by spray or grain spawn inoculation and to a lesser extent resistance to fungal spreading within the spike (type 2 resistance), assessed by single floret inoculation [95, 96]. While constitutive gene expression is expected to be unaffected by the inoculation methods we cannot exclude that the here applied single floret inoculation method was unable to detect genes that are specifically induced by Fusarium spores germinating on the spike surface and/or hyphae entering the florets which could be causal behind type 1 resistance.

## Conclusions

Infection of wheat florets by *Fg* leads to pronounced re-programming of expression patterns in several thousands of genes in the infected tissue. Though the analyzed wheat lines were chosen to represent the full range of resistance to FHB, most of the examined wheat lines share similar defense responses. The highly resistant winter wheat lines having Sumai3 as a common ancestor were distinct from the other tested lines. Generally, higher induction of gene expression in response to *Fg* was observed in the more susceptible lines with typical stress and disease response pathways being induced. The performance of Sumai3 lines may depend on several defense mechanisms associated with cell wall biosynthesis and volatile organic compound (terpene and terpenoid) emissions. These mechanisms contributed to pre-formed and/or induced resistance during the early stages of FHB infection and thus limit fungal colonization early on.

### Glossary of gene sets resulted from the analyses

CEG (Constitutively Expressed Gene): gene equally expressed under both stress (*Fg*) and control conditions

FRG (Fusarium Responsive Gene): gene significantly up- or down-regulated in response to *Fg* relative to control

DEG (Differentially Expressed Gene): gene significantly up- or down-regulated between two wheat resistance groups

C-DEG (Constitutively Differentially Expressed Gene): constitutively expressed gene significantly up- or down-regulated between two wheat resistance groups

FR-DEG (Fusarium Responsive Differentially Expressed Gene): gene significantly differentially expressed both in response to *Fg* relative to control and between two wheat resistance groups

## Supplementary Information

**Additional file 1.**

**Additional file 2.**

**Additional file 3.**

**Additional file 4.**

**Additional file 5.**

**Additional file 6.**

**Additional file 7.**

## Data Availability

The RNA-seq raw data files generated during this study are uploaded to the the NCBI BioProject. Accession number PRJNA731024 (https://www.ncbi.nlm.nih.gov/sra/PRJNA731024). The analyzed data are available as Additional files to this article.
