# Supplementary material for: Fusarium head blight resistance in European winter wheat: insights from genome-wide transcriptome analysis
Source: BMC Genomics. 2021 Jun 24;22:470. doi: 10.1186/s12864-021-07800-1 (PMC8228913; doi:10.1186/s12864-021-07800-1)
Supplement: Supplementary file 7 — Additional file 7. [file 12864_2021_7800_MOESM7_ESM.pdf]

Online Resource 7

Article title: Fusarium head blight resistance in European winter wheat: Insights from genome-wide transcriptome analysis

Journal: BMC Genomics

Authors: Maria Buerstmayr, Christian Wagner, Tetyana Nosenko, Jimmy Omony, Barbara Steiner, Thomas Nussbaumer, Klaus F.X. Mayer, Hermann Buerstmayr

Name, affiliation, and email of corresponding author:
Maria Buerstmayr, Department of Agrobiotechnology Tulln, BOKU-University of Natural Resources and Life Sciences-Vienna, Konrad Lorenz Str. 20, 3430 Tulln, Austria
e-mail: maria.buerstmayr@boku.ac.at

Figure S1 (A) Genes set enrichment analysis **(**GSEA) of genes constitutively differentially expressed (C-DEG) between Sumai3 and non-Sumai3 lines using *Fg* inoculated samples (GO biological process)**.**

Figure S1 (B) GSEA of genes between Sumai3 and Susceptible group (SUS) using mock inoculated samples (GO biological process)**.**

Figure S1 (C.1) GSEA of genes differentially expressed between Sumai3 and non-Sumai3 lines using *Fg* inoculated samples (GO biological process)**.**

Figure S1 (C.2) GSEA of genes differentially expressed between Sumai3 and non-Sumai3 lines using *Fg* inoculated samples (GO molecular function)**.**

Figure S2 Summary of enriched GO terms **(A)** per resistance group and **(B)** between pair-wise group comparison.

Figure S1 (A)

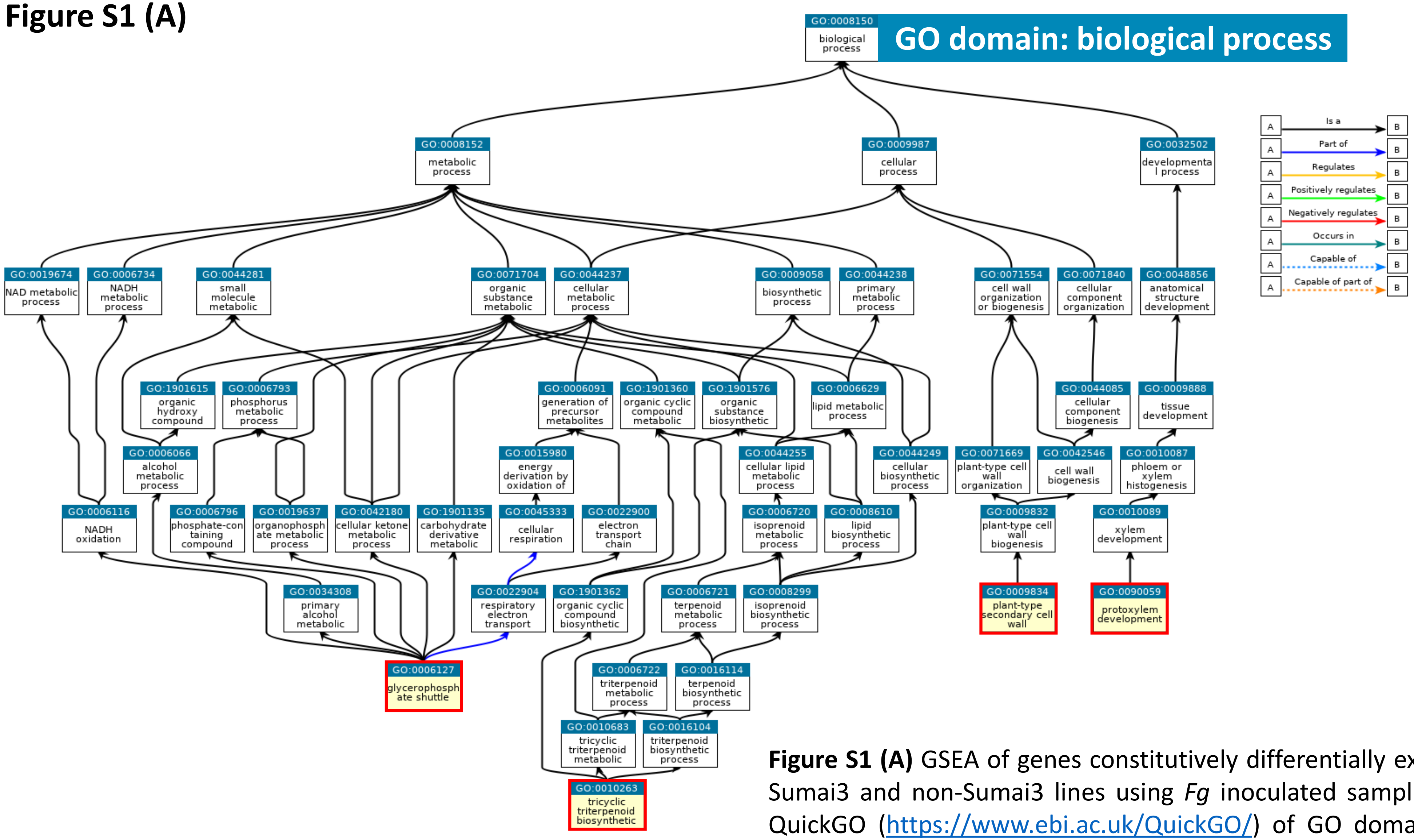

Figure S1 (A) GSEA of genes constitutively differentially expressed (C-DEG) between Sumai3 and non-Sumai3 lines using *Fg* inoculated samples. Ancestor charts from QuickGO (https://www.ebi.ac.uk/QuickGO/) of GO domain biological process. GO terms in red framed light yellow boxes are significantly enriched for genes showing higher expression levels in Sumai3 genotpyes compared to non-Sumai3 genotypes (Table S6.3).

Figure S1 (B)

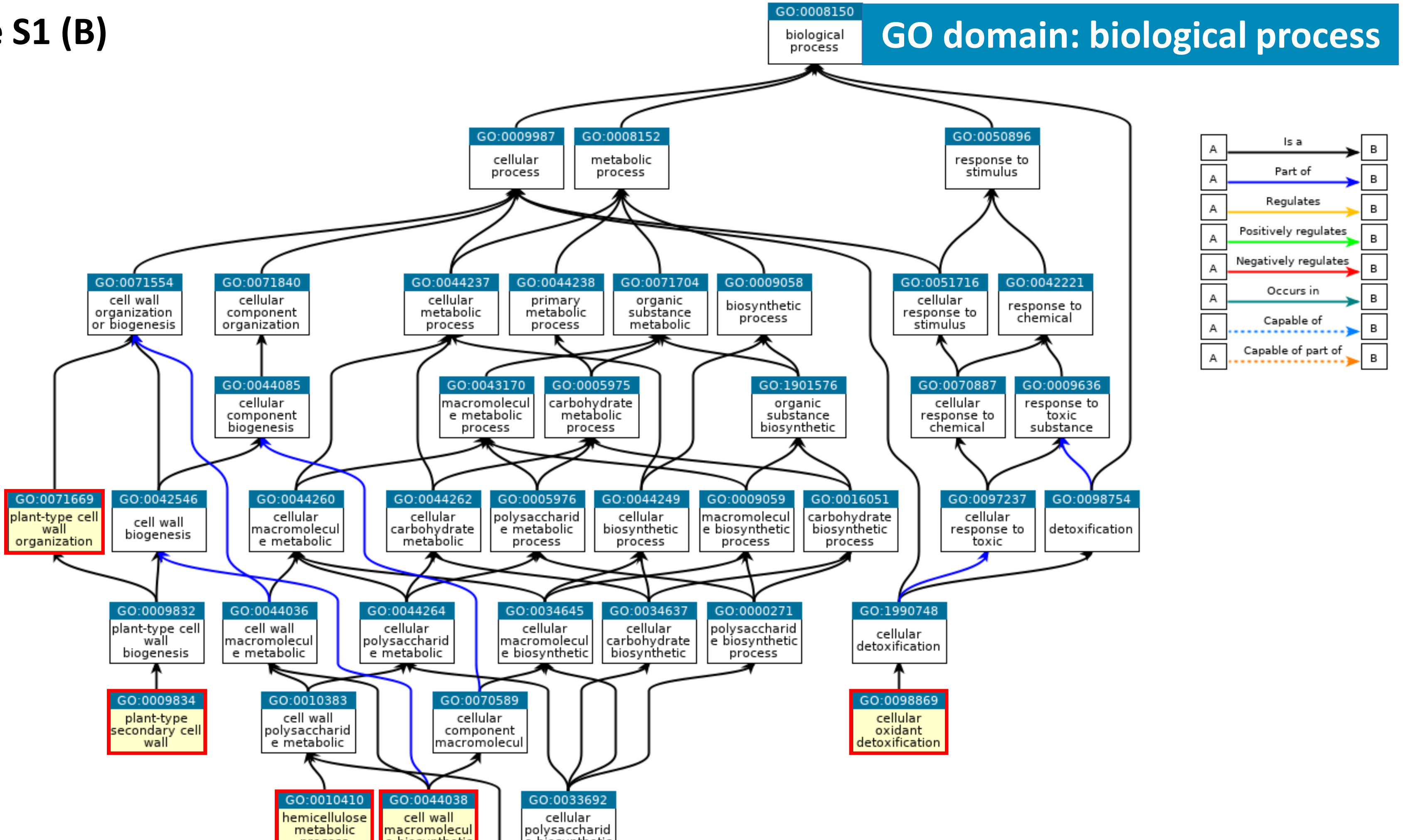

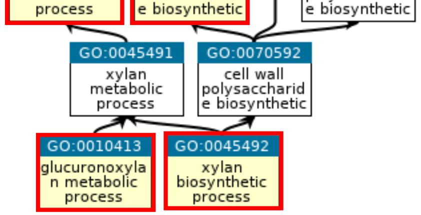

Figure S1 (B) GSEA of genes constitutively differentially expressed (C-DEG) between Sumai3 and Susceptible group (SUS) using mock inoculated samples. Ancestor charts from QuickGO (https://www.ebi.ac.uk/QuickGO/) of GO domain biological process. GO terms in red framed light yellow boxes are significantly enriched for genes showing higher expression levels in Sumai3 genotpyes compared to genotypes of resistance group SUS (Table S6.4).

Figure S1 (C.1)

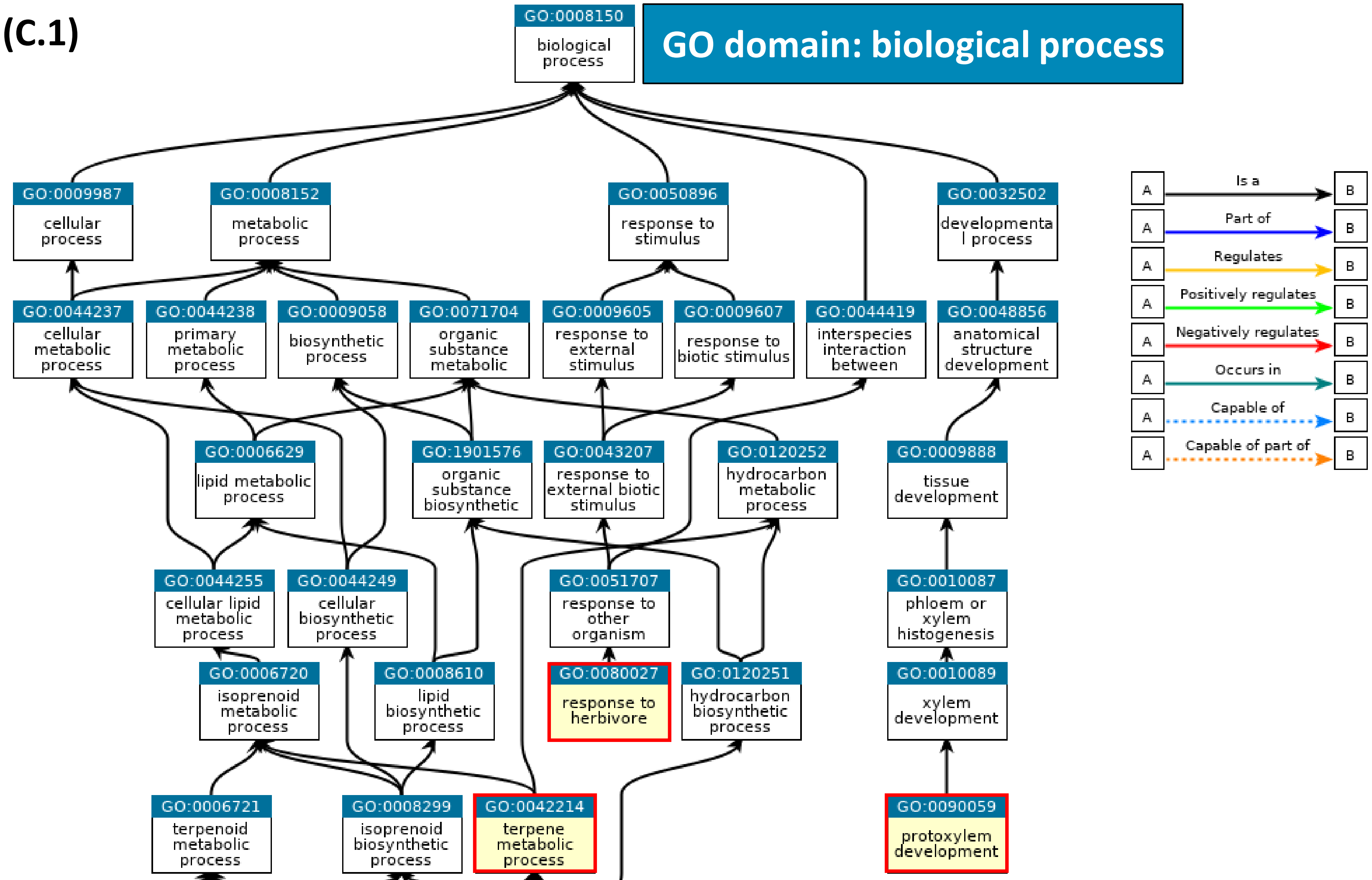

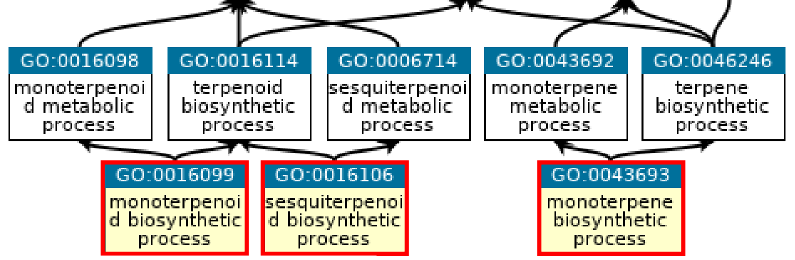

Figure S1 (C.1) GSEA of genes differentially expressed between Sumai3 and non-Sumai3 lines using *Fg* inoculated samples**.** Ancestor charts from QuickGO (https://www.ebi.ac.uk/QuickGO/) of GO domain **biological process.** GO terms in red framed light yellow boxes are significantly enriched for genes showing higher expression levels in Sumai3 genotypes compared to non-Sumai3 genotypes (see Table S6.1).

Figure S1 (C.2)

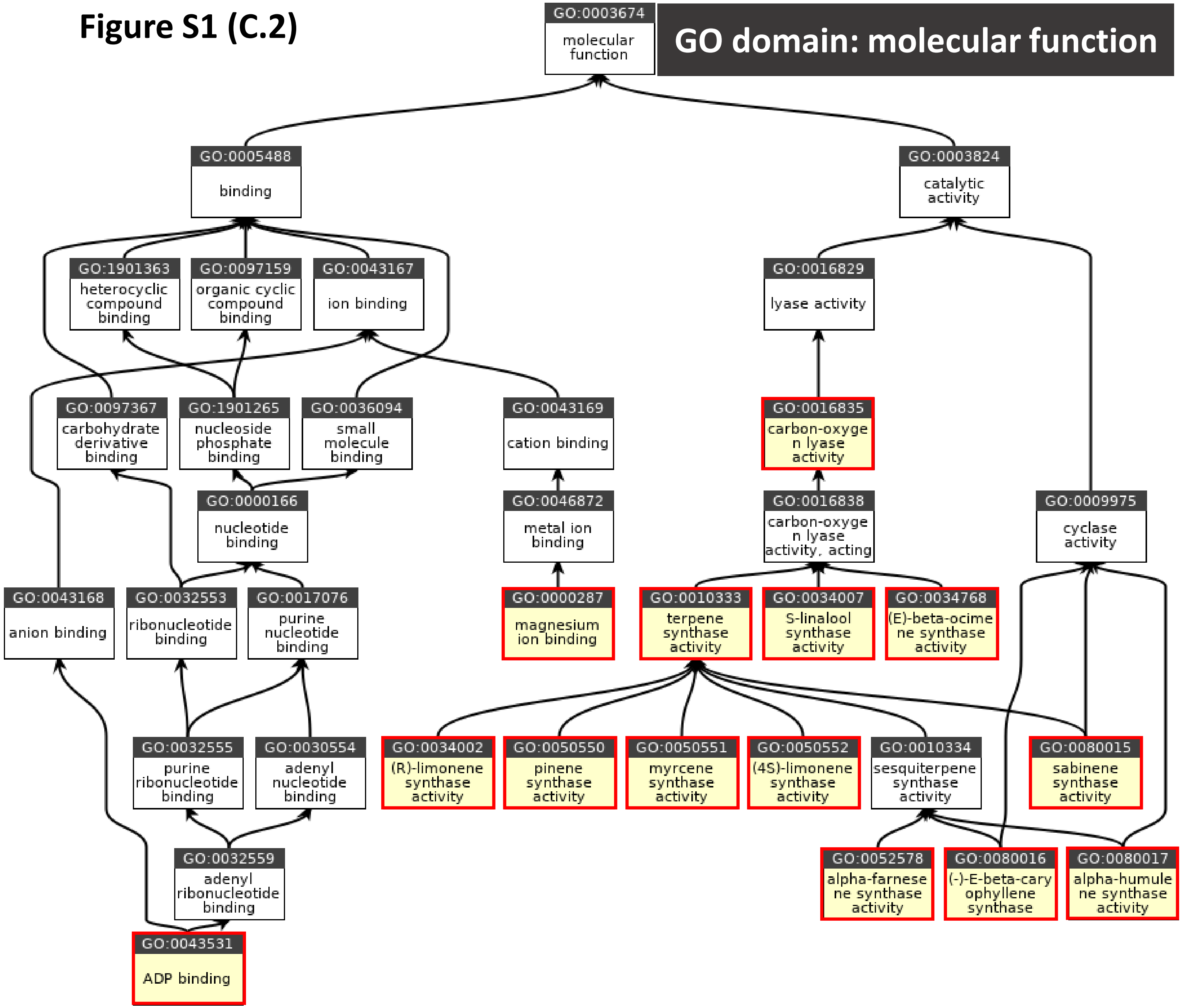

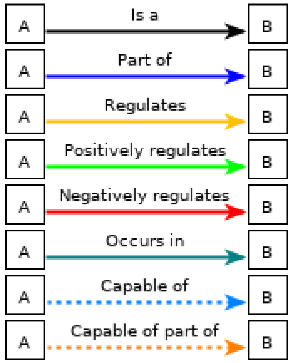

Figure S1 (C.2) GSEA of genes differentially expressed between Sumai3 and non-Sumai3 lines using *Fg* inoculated samples**.** Ancestor charts from QuickGO (https://www.ebi.ac.uk/QuickGO/) of GO domain **molecular function.** GO terms in red framed light yellow boxes are significantly enriched for genes showing higher expression levels in Sumai3 genotypes compared to non-Sumai3 genotypes (see Table S6.1).

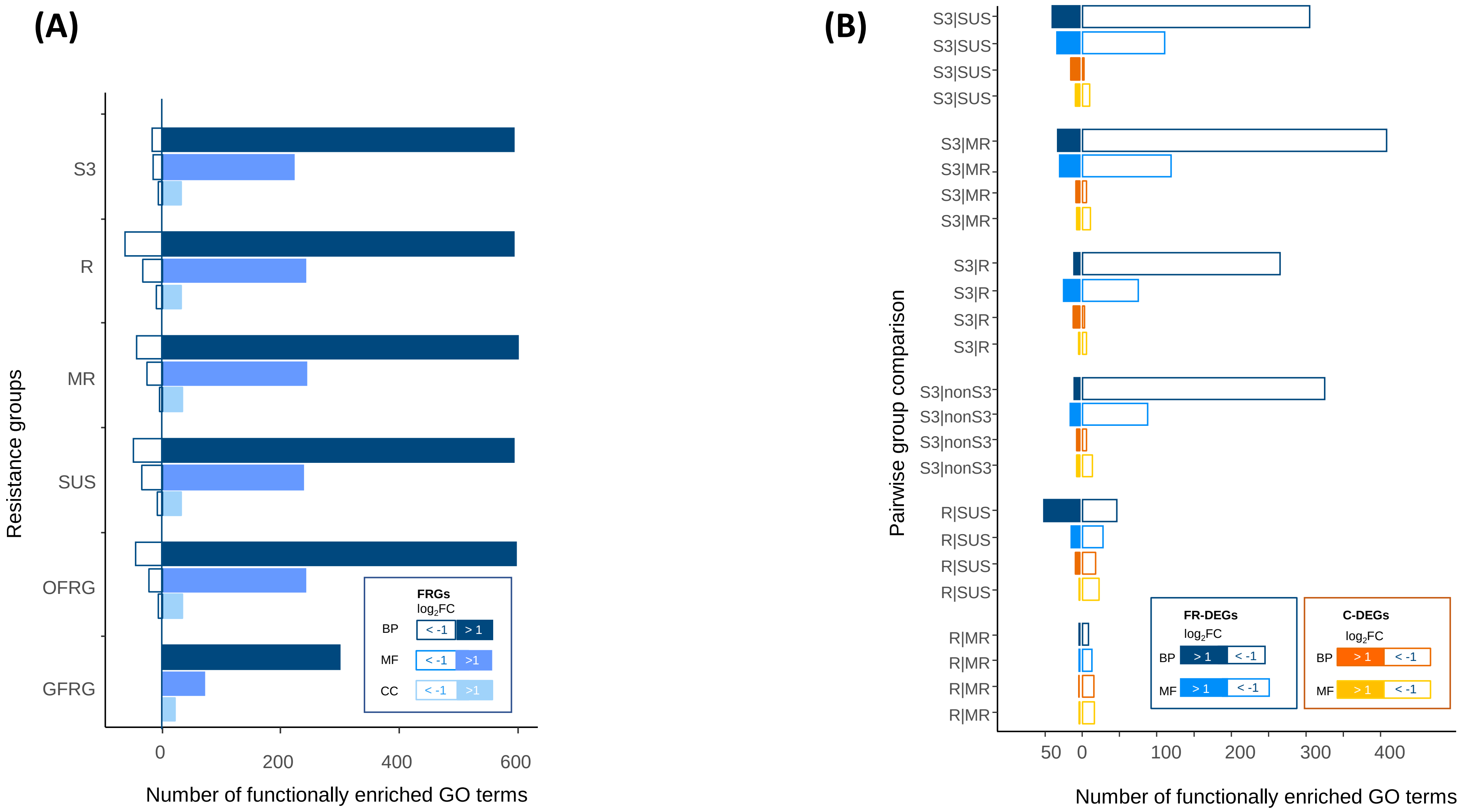

Figure S2 Number of functionally enriched GO terms of domain Biological Process (BP), Molecular Function (MF) and Cellular Component (CC) **(A)** per resistance group for up and down regulated Fusarium responsive genes (FRGs) **(B)** for genes differentially expressed between groups for FRGs (FR-DEGs) and constitutively expressed genes (C-DEGs). Groups: Sumai3 (S3), Resistant (R), Moderate Resistant (MR), Susceptible (SUS), Fusarium responsive across all genotypes [overall Fusarium responsive genes (OFRG)], Fusarium responsive in each genotype [general Fusarium responsive genes (GFRG)]
